# An evaluation of the inter-rater and intra-rater reliability of OccuPro’s functional capacity evaluation

**DOI:** 10.3233/WOR-182754

**Published:** 2018-07-31

**Authors:** Carrie Scheel, Jim Mecham, Vic Zuccarello, Ryan Mattes

**Affiliations:** a Concordia University of Wisconsin, Mequon, WI, USA; b OccuPro, LLC, Kenosha, WI, USA

**Keywords:** Return to work, workers’ compensation, kinesiophysical, industrial rehabilitation, material handling

## Abstract

**BACKGROUND:**

OccuPro’s functional capacity evaluation (FCE) is used for assessing the client’s readiness to return to work and three separate intra-rater and inter-rater reliability studies are explored here.

**OBJECTIVE:**

Three separate studies were conducted on injured and un-injured adults to evaluate the inter-rater and intra-rater reliability of the OccuPro FCE (upper extremity and material handling subtests). All three studies are summarized in this publication.

**METHODS:**

In study one, twenty participants completed firm grasp, simple grasp, pinch, fine motor, and gross motor testing. The participants included subjects with an orthopedic or musculoskeletal disorder affecting the upper extremities. In study two and three, 62 participants completed occasional squat lifts, occasional power lifts, occasional carrying, frequent squat lifts, frequent power lifts, and frequent carrying. The participants in all three studies were adult subjects between 20 and 70 years of age. Study one subjects had a previous illness or injury while subjects in study two and three had no history of injury.

**RESULTS:**

Results from study one showed that the OccuPro FCE’s four upper extremity subtests have moderate to excellent inter-rater reliability. In study two and three, results showed that the intra-rater reliability of these subtests were excellent and the inter-reliability of these subtests were moderate to good.

**CONCLUSIONS:**

These three studies establish inter-rater and intra-rater reliability for the four upper extremity subtests and material handling testing within the OccuPro FCE system. This allows for multiple therapists to use OccuPro’s FCE system with the same patient or multiple patients while having the confidence they will achieve consistent results and make sound return-to work or residual functional capacity decisions.

## 1 Introduction

Every industry has been affected by work related injuries and illnesses with a total of 1,191,100 injuries or illnesses in 2010 [1]. These injuries and illnesses cause workers to lose an average of eight days of work, resulting in a loss of wages for the worker, decreased productivity for the employer, and increased costs [1]. The economic downturn of 2009 drove increased unemployment rates with escalating medical and pharmaceutical costs due to an aging workforce, increasing obesity, and Medicare regulations, led to increased workers’ compensations costs. To reduce the rising costs of workers’ compensation, prevention strategies have been utilized to reduce risks of injuries and to ensure that injured workers are ready to return to work safely.

Occupational and physical therapists work with corporations to decrease the cost of workers’ compensation. This is achieved by evaluating each worker’s performance in the work place and implementing strategies to perform their job safely. Therapists also work with workers with injuries to assist with return to the workforce. A therapist uses Functional Capacity Evaluation (FCE) to determine the readiness of a worker with an injury to return to his or her job or to determine their residual functional capacity. The OccuPro FCE is used to determine or predict a person’s ability to return to work after suffering a work-related or non-work-related injury. The evaluation is designed to measure the functional abilities of a client, ensuring safety in their return to work.

## 2 Background

Occupational and physical therapists work with individuals who have suffered work related musculoskeletal disorders. These are muscle, nerve, or tendon illnesses or conditions occurring due to the stresses placed on the body in a work setting and at home resulting in damages to the body [2].

Prior to returning to work following a work-related or non-work-related injury, a therapist discusses job requirements, possible injury risk factors, and completes an FCE with the patient. According to Braveman and Page, a functional capacity evaluation is performed for many reasons, including working with clients to create treatment goals, making decisions about returning to work, assessing functional capacity after an injury, and deciding if an individual qualifies for disability [3]. The FCE administration includes the following: reviewing the client’s medical records, interviewing the client, screening musculoskeletal issues, assessing physical capabilities of the client, and making return to work recommendations. Demographic information, mechanism of injury, client’s employment history, client medication, baseline range of motion and strength abilities, static postures, and dynamic movements are many areas that are reviewed during the assessment [3].

Braveman and Page (2012), explain a variety of functional capacity evaluations readily available to the therapist, though many therapists choose to create their own FCE to fit their client’s specific injuries and needs [3]. However, reliability and validity have not been established for many FCEs. Portney and Watkins define reliability as “the extent to which a measurement is consistent and free from error”(p. 77) [4]. In order for clinicians to have confidence in a measurement tool, it is important to know the reliability of the tool being used. If a tool is determined to be reliable, the clinician will have more confidence in changes occurring over time, indicating real improvements/declinations in the client, rather than just an error in the measurement tool. When testing reliability, several approaches are taken to determine consistency. However, according to Innes and Straker, test-retest reliability, intra-rater reliability, and inter-rater reliability are the most common among work related assessments [5].

Two types of rater reliability are intra-rater reliability and inter-rater reliability. Intra-rater reliability refers to the consistency of the data recorded by one rater over several trials and is best determined when multiple trials are administered over a short period of time. Inter-rater reliability refers to the consistency of data recorded by two or more raters, measuring the same subjects over a single trial. Intra-rater reliability and inter-rater reliability assist in determining if a measurement tool produces results that can be used by a clinician to confidently make decisions regarding a client’s function and ability. When using an FCE tool, good reliability of that tool gives clinicians support and confidence in determining a client’s ability to return to work successfully and with little risk of re-injury [5].

## 3 Literature review

When considering inter-rater reliability for FCE’s, the higher the intraclass correlation coefficient (ICC), a statistical measure used to determine the level of reliability, the better the inter-rater reliability [6]. An excellent score of inter-rater reliability would be 0.90 to 1.00 while a good ICC score would be 0.75 to 0.90. A moderate score would be 0.50 to 0.75, and a low or poor score would be anything less than 0.50 [7, 12]. Many studies have explored the inter-rater reliability for various FCEs, including the OccuPro FCE.

Of these studies, Spanjer, et al. tested the inter-rater reliability and the validity for the Disability Assessment Structured Interview (DASI) [7]. The DASI is a semi-structured interview that assesses functional limitations in the work place by looking at impairment, activity limitations, and participation [7]. To test inter-rater reliability, two physicians administered the DASI to a single patient on the same day, but at separate times. The scores yielded by each physician were compared for each measure on the DASI. The results showed that the DASI had an intraclass correlation coefficient score of 0.81, which is considered good inter-rater reliability [7].

Gross and Battie tested the inter-rater reliability of safe maximum lifting determinants of FCE’s using the Isernhagen Work Systems for patient with low back pain [6]. Each patient was tested by a primary rater and observed by two other trained raters. The primary rater was the only rater to interact with the patient, but the other two raters also recorded scores. The scores given by the three raters were compared using an intraclass correlation coefficient. The results yielded a range from 0.95 to 0.98, showing excellent inter-rater reliability [6].

Another study by James, Mackenzie, and Capra explored the intra-rater reliability of the manual handling component of the WorkHab Functional Capacity Evaluation [8]. Four injured workers were video recorded while completing the manual handling evaluation component of the WorkHab. The manual handling component included the following three types of lifts: floor to bench lifts, bench to bench lifts, and bench to shoulder lifts. “Each lifting segment represented three lift repetitions at one weight, and a total of 35 lifting segments were included on the DVD” (p. 1798) [8]. Therapist raters scored each of the 35 lifts presented in random order, and were asked to identify whether the lift was at the individual’s maximum ability. Results were reported for each manual handling component, ranging from good to excellent with intraclass correlation coefficients ranging from 0.77 to 0.91. These findings support the WorkHab Functional Capacity Evaluation as a reliable measure to use when assessing injured workers using the manual handling subtest.

Gouttebarge, Wind, Kuijer, Sluiter, and Dresen conducted a study to determine the inter- and intra-rater reliability of the Ergo-Kit Functional Capacity Evaluation in adults without musculoskeletal complaints [9]. Twenty-seven healthy adults completed the Ergo-Kit Functional Capacity Evaluation, including all 7 Ergo-Kit tests. This was completed at three different sessions, twice by one rater, and once by another. Both raters were blind to the other’s results. Results of this study confirmed the Ergo-Kit manipulation tests have an adequate level of reliability with an intraclass correlation coefficient of 0.88 to 0.90 [9].

Tuckwell, Straker and Barret in 2002 studied the test-retest reliability of nine tasks of the ErgoScience Physical Work Performance Evaluation. These nine tasks were primarily associated with dynamic strength, positional tolerance and mobility. “The tasks of kneeling, lifting floor to waist, bi-lateral carry and pushing were found to have substantial test-retest reliability; standing and repetitive squatting moderate to substantial test-retest reliability; the sitting and walking tasks were found to have fair to moderate reliability and stair climbing task was fond to have only slight reliability due to error” [14].

OccuPro’s FCE was developed as a tool to evaluate and predict injured worker’s ability to successfully return to work without re-injury. To assure that clinicians can have confidence in this tool, it was important to evaluate the inter-rater and intra-rater reliability of the OccuPro FCE. The purpose of this publication is to investigate three different research projects performed by Occupational Therapy students at Concordia University Wisconsin who studied the inter-rater and intra-rater reliability of the OccuPro FCE with injured and healthy workers. It is important that the OccuPro FCE methodologies have acceptable inter-rater and intra-rater reliability so that clinicians may have confidence that the tool will remain accurate over time when measured and re-measured by one clinician or by multiple different clinicians.

## 4 Methods

### 4.1 Design

It is important that research performed on medical testing is done independently from the developer of the medical test to reduce any potential bias. The developer of OccuPro’s FCE methodology, Jim Mecham, MSIE, OTR/L, CPE, provided standard FCE performance training at Concordia University of Wisconsin to students in the Occupational Therapy and Rehabilitation Science Programs who were working towards their Master’s degrees. All three research projects summarized in this publication where carried out from beginning to thesis exclusively by Occupational and Physical Therapy students at Concordia University Wisconsin, overseen by Carrie Scheel, EdD, OTR/L, CPE. All three research projects where approved by Concordia University Wisconsin’s Institutional Review Board prior to data collection.

In study one, Buckley, Ferracane, and Pickerill analyzed the inter-rater reliability of upper extremity testing within the OccuPro FCE and whether an FCE examiner could consistently determine occasional, frequent or constant return to work levels [13]. In study two, Devaguptapu used an exploratory design investigating the intra-rater reliability of occasional and frequent material handling testing within the OccuPro FCE methodology [10]. In the third study, Gunda investigated the inter-rater reliability of occasional and frequent material handling also within the OccuPro FCE [11].

### 4.2 Participants

Study one, consisted of 27 subjects who were recruited from a local physical therapy clinic by treating Occupational Therapists who asked their existing upper extremity patients if they were interested in participating in a research project [13]. They all were identified as having an orthopedic or musculoskeletal disorder affecting the upper extremities. Subjects that underwent recent surgery (within 8 weeks) or an acute injury were excluded from the study. All participants were between the ages of 18 and 75. Seven subjects were unable to complete all three data collections. Reasoning for exclusion of these subjects included missed sessions (vacation, illness, etc.) and 2 subjects chose not to participate after reading the consent form. The final study sample consisted of 20 subjects.

Study two and three respectively included 31 healthy adults with no history of current or previous illness or injury [10, 11]. Seventeen females and 14 males were chosen from enrolled students at Concordia University Wisconsin using convenience sampling. Participants were between 20 and 70 years of age. Exclusion criteria for these studies included: participants not within the age range, injured workers, and participants with medical impairments. Three subjects were unable to complete the study for various reasons. The final study sample consisted of 28 subjects.

### 4.3 Procedures

In study one, Buckley, Ferracane, and Pickerill analyzed upper extremity subtests of the OccuPro FCE [13]. Prior to testing, three separate raters from the Master of Occupational Therapy Program at Concordia University Wisconsin were trained in a four-hour FCE procedure course focusing on the upper extremity subtests of the OccuPro FCE. All testing was completed by these trained raters. Upper extremity subtests included evaluation of simple and firm grasp, pinch, gross motor, and fine motor. Five participants were randomly assigned to each rater during the first session. The participants then rotated to a different rater during the next two sessions, allowing each participant to be individually tested by each of the trained raters over a two-week period. Each session took about 30 minutes to complete. This process was completed a total of three times over a six week period of time. Between data collection sessions, the participants performed their normal daily activities.

The four OccuPro FCE upper extremity subtests were conducted using different measurement tools. In order to reduce incidents of fatigue and irritation of current injury, participants were offered a one minute break between subtests. The grasp strength assessment was measured using a grip strength dynamometer. Three measurements were taken for each hand and the mean was compared with the Mathiowetz age and gender norms. Pinch strength was measured using a pinch dynamometer. Three different pinch measurements were assessed including lateral pinch, palmar pinch, and tip pinch. Three measurements were taken for each hand and the mean was compared with the Mathiowetz age and gender norms. Gross motor was measured using the Box and Blocks assessment. Both hands were assessed and a total number of blocks were counted for each trial and compared to Matheowitz age and gender norms. Fine motor was assessed using the Purdue Peg Board assessment. Both hands were assessed with four total trials and compared to the Purdue Peg Board norms. Biomechanics were analyzed, a functional pain score gathered, pain behaviors noted, and a comparison to their demonstrated functional ability was performed after each of the subtests. Table 1 shows the decision-making process using OccuPro’s decision making algorithms.

**Table 1:** OccuPro Decision Charts

		Avoid	Occasional	Frequent	Constant
Simple Grasping	Ability	Client is unable to generate any poundage on dynamometer	Client able to generate some poundage on dynamometer	Client able to achieve grip strength that is within normal age and gender ranges per Matheowitz norms	Male clients are able to achieve grip strength that is within 21 points of their mean and female clients are within 12 points of their mean or greater per Matheowitz age and gender norms
	Mechanics	Clientdemonstratesmechanical deficits to include but not limited to significant loss of distal upper extremity range of motion.	Client demonstrates mechanical changes to include but not limited to distal upper extremity range of motion.	Client demonstrates no loss of distal upper extremity mechanics	Client demonstrates no loss of distal upper extremity mechanics
	Functional Pain	Client understands and appropriately uses the OccuPro pain intensity scale, has minimal to no reliability of pain issues and reports increases in pain symptoms toa6or higher specifically associated with the distal upper extremity.	Client understands and appropriately uses the OccuPro pain intensity scale, has minimal to no reliability of pain issues, and reports pain at 5 or less specifically associated with the distal upper extremity.	Client understands and appropriately uses the OccuPro pain intensity scale, has minimal to no reliability of pain issues and reports pain at a 4 or less specifically associated with the distal upper extremity.	Client understands and appropriately uses the OccuPro pain intensity scale, has minimal to no reliability of pain issues and reports pain symptoms at a 2.5 or less specifically associated with the distal upper extremity.
	Pain Behav¬iors	Client exhibits significant facial grimacing, verbal grunting, holding, and/or requires a break following this test and is specifically associated with pain in the distal upper extremity.	Client exhibits mild pain behaviors including but not limited to facial grimacing and is specifically associated with pain in the distal upper extremity.	Client exhibits no pain behaviors specifically associated with pain in the distal upper extremity.	Client exhibits no pain behaviors specifically associated with pain in the distal upper extremity.
	Functional Demon¬stration	Client exhibits an inability to perform material handling activities secondary to functional grip strength	Client exhibited the ability to perform occasional material handling activities at low weight levels	Client exhibited the ability to perform all occasional and frequent material handling activities at low weight levels	Client exhibited the ability to perform all material handling activities without deficit

In study two, Devaguptapu had healthy subjects perform occasional and frequent lifting and carrying testing [10]. The material handling tests were assessed by the same rater each time to establish intra-rater-reliability with the second rating being performed within two weeks of the first rating. Each subject was asked to perform an occasional and frequent bilateral squat lift, power lift and carry using a kinesiophysical approach. Occasional material handing testing was performed starting at low weight levels with one repetition at each weight increment and progressing up to a level where the subject exhibited biomechanical deficits. The weight level was then lowered by 5 or 10 pounds to the safe occasional lift/carry. Frequent material handling was tested starting at a low weight levels and progressed using a five-repetition approach and progressing up to the subject’s safe biomechanical change (single substitution pattern) level. The weight level was then lowered by 5 or 10 pounds to establish the safe frequent material handling level. The subject’s heart rate and rating of perceived exertion were measured with a heart rater monitoring strap and Borg Rating of Perceived Exertion Scale at the end of each task. Subjects were informed to stop the test at any time if they felt unsafe or their maximum capacity performance was reached prior to the rater stopping the test.

In study three, Gunda had healthy subjects perform occasional and frequent lifting and carrying testing with two different raters to establish inter-rater reliability with the second rater performing their data collection within two weeks for the first rater’s data collection [11]. Each subject was asked to perform an occasional and frequent bilateral squat lift, power lift and carry using a kinesiophysical approach. Occasional material handing testing was performed starting at low weight levels with one repetition at each weight increment and progressing up to a level where the subject exhibited biomechanical deficits. The weight level was then lowered by 5 or 10 pounds to the safe occasional lift/carry. Frequent material handling was tested starting at a low weight levels and progressed using a five-repetition approach and progressing up to the subject’s safe biomechanical change (single substitution pattern) level. The weight level was then lowered by 5 or 10 pounds to establish the safe frequent material handling level. The subject’s heart rate and rating of perceived exertion were measured with a heart rater monitoring strap and Borg Rating of Perceived Exertion Scale at the end of each task. Subjects were informed to stop the test at any time if they felt unsafe or their maximum capacity performance was reached prior to the rater stopping the test.

### 4.4 Data analysis

In all three studies the Intraclass Correlation Coefficient (ICC) was used to measure the consistency of therapist ratings for FCE subtests and was calculated using SPSSv.20. Koo and Li in 2016 classified the ICC as follows: values less than 0.50 are considered poor reliability, 0.50 to 0.75 are considered moderate reliability, 0.75 to 0.90 is considered good reliability and ICC’s greater than 0.90 is considered excellent reliability [12].

In study one, Buckley, Ferracane, and Pickerill studied the inter-rater reliability of the upper extremity subtests of the OccuPro FCE [13]. Data collected included whether the person should avoid the upper extremity subtest or if they could perform the subtests occasionally, frequently or constantly as it relates to work.

In study two, Devaguptapu measured the intra-rater reliability of the OccuPro FCE by measuring intra-class correlation coefficients (ICC) between 28 subjects [10]. The assessments included 6 subtests that were measured by the same rater twice over a period of 2 weeks. There are six models for calculating the ICC. Model 3 was chosen for this research study because each subject was assessed by each of the two raters and these were the only raters available. Because there were only two raters, random selection of raters was not applicable. ICC scores range from 0.00 to 1.00, with values above 0.75 representing good to excellent reliability, and values below 0.75 representing poor to moderate reliability.

In study three, Gunda measured the inter-rater reliability of the OccuPro FCE by completing the intra-class correlation coefficient (ICC) between 28 subjects [11]. The assessment included 6 subtests that were measured by two different raters. The second model of the ICC was used to assess inter-rater reliability.

## 5 Results

Following participant dropout in the first study, 20 subjects were able to complete all three data collection sessions [13]. Data analysis yielded ICC values ranging from 0.656 to 0.931. The study revealed the ICC for firm grasp (R = 0.916), simple grasp (R = 0.828), pinch (R = 0.656), fine motor (R = 0.756), and gross motor (R = 0.931). Overall the ICC reliability for study one showed moderate to excellent reliability and all ICC’s were statistically significant (*p* = 0.05) (Table 2).

**Table 2:** Inter-rater reliability

Subtest	ICC Value	*P* Value
Firm Grasping	0.916	^*^0.000
Simple Grasping	0.828	^*^0.000
Pinching	0.656	^*^0.003
Fine Motor Coordination	0.756	^*^0.000
Gross Motor Coordination	0.931	^*^0.000
Occasional Squat Lift	0.804	Not Reported
Frequent Squat Lift	0.869	Not Reported
Occasional Power Lift	0.738	Not Reported
Frequent Power Lift	0.747	Not Reported
Occasional Carrying	0.804	Not Reported
Frequent Carrying	0.849	Not Reported

^*^P value is statistically significant (<0.05).

The ICC analysis for the second study revealed the intra-rater reliability for occasional squat lift (R = 0.948), occasional power lift (R = 0.975), occasional carrying (R = 0.924), frequent squat lift (R = 0.854), frequent power lift (R = 0.932), and frequent carrying (R = 0.927). Overall ICC reliability for these subtests ranged from 0.85 to 0.97, which showed good to excellent intra-rater reliability (Table 3).

**Table 3:** Intra-rater reliability

Subtest	ICC Value	*P* Value
Occasional Squat Lift	0.948	^*^0.000
Frequent Squat Lift	0.854	^*^0.000
Occasional Power Lift	0.975	^*^0.000
Frequent Power Lift	0.932	^*^0.000
Occasional Carrying	0.924	^*^0.000
Frequent Carrying	0.927	^*^0.000

^*^P value is statistically significant (<0.05).

The results related to inter-rater reliability for study three, revealed the ICC for occasional squat lift (R = 0.804), occasional power lift (R = 0.738), occasional carrying (R = 0.804), frequent squat lift (R = 0.869), frequent power lift (R = 0.747), andfrequent carrying (R = 0.849) [11]. The ICC of these subtests ranged from 0.66 to 0.86, which showed moderate to good inter-rater reliability (Table 2).

## 6 Discussion

### 6.1 Main findings

In study one, Buckley, Ferracane, and Pickerill, established the inter-rater reliability of the upper extremity subtests of the OccuPro FCE [13]. One of the challenges for FCE’s as a whole has been an examiner making a decision of the ability of the worker to perform at an occasional, frequent or constant level following a standardized test of grasping, pinching, fine motor coordination and gross motor coordination. The results of study one show that the firm grasp, simple grasp, fine motor, and gross motor subtests have good to excellent inter-rater reliability and that multiple testers can have the confidence that on the same patient they would come to the same decisions on whether this patient can perform grasping and coordination on an occasional, frequent or constant basis in regards to work. The results of the pinch subtest showed moderate inter-rater reliability and was noted to have the lowest ICC score within the first study. The decision-making procedures within the OccuPro FCE has been praised by users throughout the world as a means of having a brand new FCE examiner and an experienced FCE examiner come to similar consistent conclusions related to return-to-work decisions. The pinch testing inter-rater reliability was moderate which shows the return to work decision-making procedures for the pinch testing subtests could be defined better so the FCE examiner can make consistent return to work decisions.

Devaguptapu, in study two showed good to excellent intra-rater reliability when studying occasional and frequent lifting and carrying [10]. One of the primary decisions when determining return to work comes from the physical demand level of occasional, frequent, or constant material handling. It is tied tightly into the US Department of Labor Physical Demand Categories (PDC) which is defined in the Dictionary of Occupational Titles. Within the various FCE testing methodologies on the market there are slightly different methodologies to determine safe occasional and frequent material handling levels. FCE examiners using the OccuPro FCE testing method can have the confidence that the same examiner will make reliable decisions on the same patient if asked to perform multiple FCE’s on a patient.

Study three performed by Gunda showed moderate to good inter-rater reliability between occasional and frequent lifting and carrying [11]. It could be argued that inter-rater reliability, where two separate raters come to the same conclusion on one patient, has a higher level of importance within an FCE. It is noted that floor to waist lifting and bilateral carrying have good reliability which again tend to be the primary decision making parameters to determine the Physical Demand Category (PDC) in an FCE. The 12 inch to waist lift, both occasionally and frequently, had moderate reliability. This level of reliability allows FCE examiners to have confidence that separate raters make reliable decisions about occasional and frequent material handling and subsequently reliable Sedentary, Light, Medium, Heavy or Very Heavy PDC level decisions.

### 6.2 Limitations

In study one, a limitation was the sample size secondary to a high attrition rate. Twenty seven subjects were originally recruited for the study but only 20 participated in all three data collection sessions. A larger sample size would increase the confidence in the results and allow for greater precision [13]. A further limitation was the written decision-making directions provided for the pinch and fine motor subtests. Following the performance of a functional test, the decision-making procedures are read by the FCE examiner and there was some ambiguity associated with pain ratings. Following a functional test, a testing subject reports a level of pain on a 0–10 functionally based pain scale and some raters scored the examinee higher and some scored them lower in regards to return to work ability secondary to the written directions. An improvement to the pain level verbiage in the decision-making procedures may help the reliability of pinch testing and fine motor coordination. Some further limitations outlined by the researchers included the examinees having lotion on their hands and fatigue as testing progressed.

In study two and three, a limitation to each of these studies was that all participants in the study were un-injured, healthy individuals and a sample of convenience. Although several research projects within FCE literature performed reliability research on healthy subjects, there is more value in FCE research when the study is performed on injured subjects since this has more similarity to an actual FCE. A further limitation to study two and three was that healthy university students were tested performing maximal lifting and carrying. Many of them reported that on session one they performed at their maximum level as requested and were sore the following day. This is like the soreness a patient might experience from an actual FCE in a clinic. However, the examinees during the second round of material handling testing verbally reported they did not want to lift and carry as much as they did on the first day of data collection as they did not want to be as sore as they were the first time. The examiners, as would be standard in an FCE, allowed the university students to stop the test if they chose to stop the test.

A limitation associated with the compilation of studies in this singular body of work include the separate and distinct variables that were measured in each study. Combining the three separate studies into one body of work limited the ability to combine variables which would have enhanced statistical power. Furthermore, the authors were unable to perform higher level statistical analysis as one overall study due to the different variables measured in each of the three studies.

Future research on the OccuPro FCE should include additional components of the FCE subtests, use of injured subjects, and larger sample sizes to improve the statistical power of the research.

## 7 Conclusion

In the first study conducted on the OccuPro FCE, the researchers found the upper extremity subtests which included grasping, pinching, fine motor coordination, and gross motor coordination to have moderate to excellent inter-rater reliability with subjects that had suffered an upper extremity injury [13]. In the second study the overall ICC scores for the OccuPro FCE revealed good to excellent intra-rater reliability for occasional and frequent material handling [10]. The third study completed on the OccuPro FCE testing system showed ICC scores for inter-rater reliability of uninjured subjects having moderate to good inter-rater reliability for occasional and frequent material handling testing [11]. The results of all three of these studies provide clinicians confidence in the use of the OccuPro Functional Capacity Evaluation system and its ability to provide consistent results between multiple patients, between the same FCE examiner, and among multiple FCE examiners. The thousands of professionals who use the OccuPro FCE testing methodology all over the world can have the confidence that they are producing credible functional results to their referral sources and making sound decisions in regards to return to work and a client’s residual functional capacity.

## Conflict of interest

None to report.
